# Diversity, Composition and Functional Inference of Gut Microbiota in Indian Cabbage white *Pieris canidia* (Lepidoptera: Pieridae)

**DOI:** 10.3390/life10110254

**Published:** 2020-10-25

**Authors:** Ying Wang, Jianqing Zhu, Jie Fang, Li Shen, Shuojia Ma, Zimiao Zhao, Weidong Yu, Weibin Jiang

**Affiliations:** 1 Laboratory of Environmental Entomology, College of Life Sciences, Shanghai Normal University, 100 Guilin Rd., Shanghai 200234, China; 1000460649@smail.shnu.edu.cn (Y.W.); 1000479678@smail.shnu.edu.cn (J.F.); 1000479682@smail.shnu.edu.cn (L.S.); 1000459151@smail.shnu.edu.cn (S.M.); 1000459159@smail.shnu.edu.cn (Z.Z.); ywd@shnu.edu.cn (W.Y.); 2 Shanghai Zoological Park, 2381 Hongqiao Rd., Shanghai 200335, China; zzzjjq@gmail.com

**Keywords:** Indian cabbage white, gut microbiome, 16S rDNA sequencing, developmental stage

## Abstract

We characterized the gut microbial composition and relative abundance of gut bacteria in the larvae and adults of *Pieris canidia* by 16S rRNA gene sequencing. The gut microbiota structure was similar across the life stages and sexes. The comparative functional analysis on *P. canidia* bacterial communities with PICRUSt showed the enrichment of several pathways including those for energy metabolism, immune system, digestive system, xenobiotics biodegradation, transport, cell growth and death. The parameters often used as a proxy of insect fitness (development time, pupation rate, emergence rate, adult survival rate and weight of 5th instars larvae) showed a significant difference between treatment group and untreated group and point to potential fitness advantages with the gut microbiomes in *P. canidia*. These data provide an overall view of the bacterial community across the life stages and sexes in *P. canidia*.

## 1. Introduction

*Pieris canidia* (Sparrman, 1768), the Indian cabbage white, is a major pest of crucifer plants in several countries of Asia [1]. It consumes leaf tissue, causes deficit disease and reduces production of crops. The chemical insecticide is the main control method at present, but it results in the increase of resistance to the pesticide, ecological environment deterioration and human health impaired with pesticide residues [2]. Thus, the development of bio-control techniques for agricultural pests is a research prospect for pollution-free vegetable production [3].

In the process of evolution, the gut microbiomes of insects interact and co-evolve with their hosts [4]. The gut microbiota plays crucial roles in the growth, development and immunity of the host insects [5]. The correlation between gut microbiota of the insect and the resistance of chemical insecticide provides a new perspective for comprehensive control of agricultural pests. The relationship between gut microbiomes and feeding behavior of their hosts also provides a theoretical model for insect evolution studies. Furthermore, the interaction between gut microbiomes and host immune systems is an ideal model for the study of insect immune mechanisms, which contributes to the biological control of pests and development of pesticide targets [6].

Hammer et al. [7] characterized the gut microbiomes of 185 wild lepidopteran caterpillars from 124 species of 15 families with 16S rRNA gene sequencing and quantitative PCR. The microbes detected in caterpillar guts were low-density and variable among individuals compared with other insects and vertebrates. In nearly 300 adult butterflies representing over 50 species from six families, Ravenscraft et al. [8] did not find a lot of bacteria load of gut microbiomes but indicated that the majority of variation in the butterfly gut flora is expressed at the level of the individual host, followed by host species. Their results show that caterpillars lack a resident gut microbiome, and the gut microbial community composition varies across host individuals.

In this study, we characterized gut microbial abundance and composition of the larvae and adults via 16S rRNA gene sequencing and observed the development and fitness of butterflies treated with antibiotics. We laid the foundation for a mechanistic understanding of how this hidden symbiosis affects and is affected by its host. 

## 2. Materials and Methods

### 2.1. Sampling, Sequencing and Bioinformatics Analyses

The *P. canidia* butterflies used in this study were collected from three wild populations (Xuhui N 31.22° E 121.48°, Songjiang N 31.00° E 121.24° and Chongming N 31.73° E 121.40°) in Shanghai, China in June 2019. The adults were caught with a sweep net in the field, and the larvae were found on their host plants *Brassica campestris* L. We collected twenty adults (10 females and 10 males) and twenty of the last instar larvae (10 females and 10 males) in each local population. These insects were dissected and the gut samples preserved with dry storage at −20 °C. The storing method for insect microbiome samples does not substantially alter community composition [9]. Each ten dissected tissues of a same group within each population were pooled as a sample to extract DNA. We extracted DNA with a Powersoil DNA isolation kit and protocol (MoBio, CA, USA). The 16S rRNA V3-V4 gene section was conducted with PCR amplification with barcoded primers 357F (5′-ACTCCTACGGRAGGCAGCAG-3′) and 806R (5′-GGACTACNVGGGTWTCTAAT-3′) and the methods from Liu et al. [10]. Libraries were pooled, and 450-bp paired end reads were sequenced on an Illumina MiSeq sequencer.

The paired-end fastq files of 16S rRNA amplicons generated by Illumina were used as input files. Sequences were pre-processed, quality filtered and clustered into operational taxonomic units (OTU) at the 97% sequence similarity level using UPARSE [11]. They were annotated and classified using the Ribosomal Database Project (RDP) classifier and Greengenes [12,13]. We used one-way ANOVA to determine whether there were significant differences in community richness or the relative abundances of individual bacterial taxa.

Predicted functions of *P. canidia* bacterial communities were inferred using PICRUSt [14]. The analysis was performed by the predict_metagenomes.py script run against the functional database of KEGG Orthology. Functional contributions of various taxa to different KOs were computed with the script metagenome_contributions.py [15,16]. 

### 2.2. Antibiotic Experiment

We collected *P. canidia* eggs of a Xuhui population (N 31.22° E 121.48°) from *B. campestris* plants. Two hundred and forty newly hatched larvae were randomly and evenly divided into three tetracycline treatment groups (0.56 mg/mL, 1.12 mg/mL and 1.68 mg/mL) and a control group. The antibiotics used here have been shown to suppress bacterial symbionts in other insect [17]. Water with or without antibiotics was sprayed onto leaves, which were then briefly dried before feeding according to Hammer et al. [7]. Each group had six biological replicates and each replicate included 10 larvae. Each replicate was reared in a separate plastic case (dimensions of 15 cm × 15 cm × 10 cm) on *B. oleracea* foliage and transferred to a butterfly cage (dimensions of 28 cm × 28 cm × 30 cm) when they were 5th instars.

We compared development time, pupation rate, emergence rate, adult survival rate and weight of 5th instars larvae between tetracycline treated (0.56 mg/mL, 1.12 mg/mL and 1.68 mg/mL) and untreated groups by one-way ANOVA. These parameters are often used as a proxy of insect fitness [18,19]. All analyses were conducted using SPSS statistics version 21.0 for Windows (SPSS Inc., Chicago, IL, USA).

## 3. Results

### 3.1. Bacterial Community Diversity and Predicted Functional Metagenomes in P. canidia

**Figure 1:**
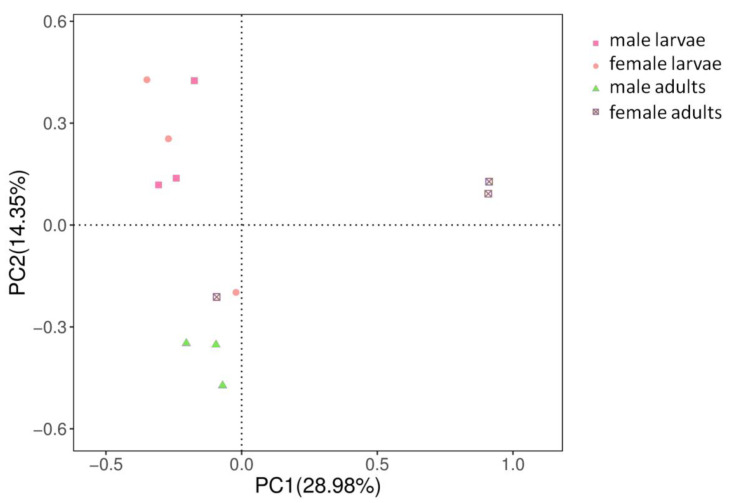
Principal component analysis based on operational taxonomic units (OTUs). X-axis, 1st principal component; Y-axis, 2nd principal component. Numbers in parentheses represent the contributions of the principal components to differences among samples. Dots represents individual samples, and different colors represent different groups.

The raw sequence data obtained in this study were deposited in The National Center for Biotechnology Information (NCBI) under accession number SRS5823888-SRS5823899. Raw sequences clustered into 509 operational taxonomic units (OTUs) across the 12 samples (Table 1). These OTUs were annotated into 12 phyla, 24 classes, 45 orders, 78 families and 120 genera (Table 2), of which 39 OTUs were shared across all groups (Supplementary Materials Figure S1) and 4 OTUs were shared across all samples (Figure S2). Sufficient sequencing data were obtained based on the plateaued rarefaction curves of obvious species (Figure S3). Based on the OTU abundance information (97% similarity), the relative abundance of each OTU in each sample was calculated, and the PCA (principal component analysis) of OTU was done with the relative abundance value (Figure 1). Coordinate dots of female larvae and male larvae samples were closely located.

**Figure 2:**
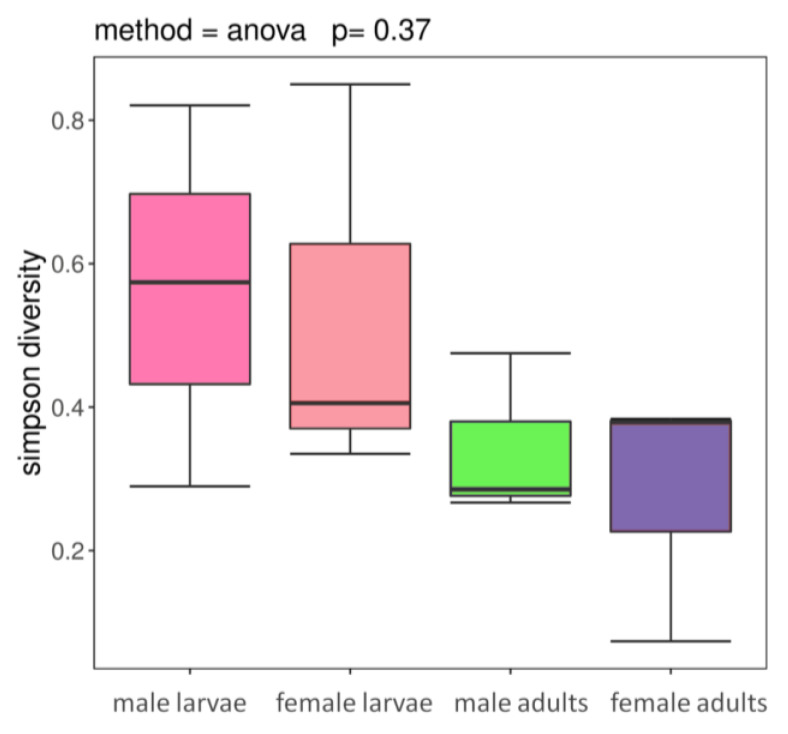
Changes in bacterial community diversity across life stages and sexes. Boxplot of Simpson Diversity Index values from *P. canidia* male larvae, female larvae, male adults and female adults.

Bacterial phylotype richness did not show a significant difference among life stages and sexes (F _(3,8)_ = 0.013, *p* = 0.998). Nearly identical patterns were observed when diversity was measured using the Simpson index (Figure 2), which takes relative abundances into account (F _(3,8)_ = 1.196, *p* = 0.371). A comparison restricted to only the numerically dominant phylotypes (at least 1%) produced a similar pattern (F _(3,8)_ = 2.628, *p* = 0.122). Gut bacteria of larval samples were more diversified than those of adult samples, which were derived from five diversity estimators in Table 3. Higher value of observed species (sobs), Chao, Ace, Simpson’s index and lower Shannon’s index in male larvae and female larvae groups suggested that gut bacteria from larval guts were more diverse than those from the adult stage.

**Figure 3:**
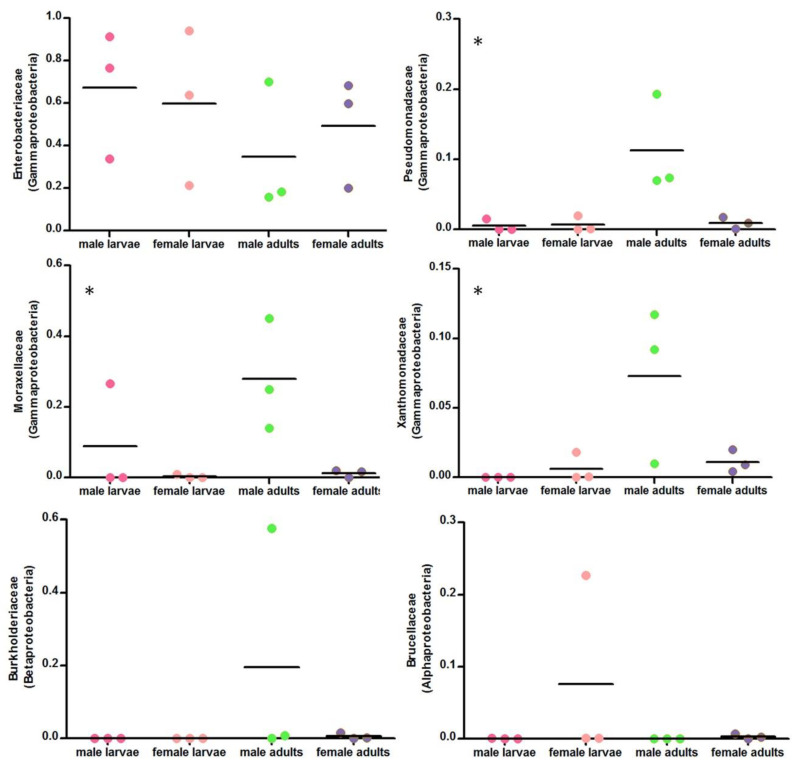
Dynamics of bacterial families across life stages and sexes. Relative abundances of the six dominant bacterial families in *P. canidia* were displayed. Points show three individual values. Bars show average relative abundances. Asterisks indicate bacterial families whose relative abundances differed significantly across life stages (* *p* < 0.05, ** *p* < 0.01).

The four groups of *P. canidia* (male larvae, female larvae, male adults and female adults) were dominated by six bacterial families: the Enterobacteriaceae and Moraxellaceae (Gammaproteobacteria), Burkholderiaceae (Betaproteobacteria), Pseudomonadaceae and Xanthomonadaceae (Gammaproteobacteria), and Brucellaceae (Alphaproteobacteria). Although family-level bacterial community composition varied substantially between individuals of the same life stage in some cases, three families including Moraxellaceae, Pseudomonadaceae and Xanthomonadaceae shifted significantly in relative abundance across the life stages and sexes (Figure 3, Bonferroni-corrected, *p* < 0.05). The 20 most abundant phylotypes present across all four groups of *P. canidia* samples are listed in Table 1. The split between larval and adult communities appears to be driven by the higher relative abundance of *Kluyvera* in the larvae and of *Acinetobacter guillouiae* and *Stenotrophomonas* in the mature adults.

**Figure 4:**
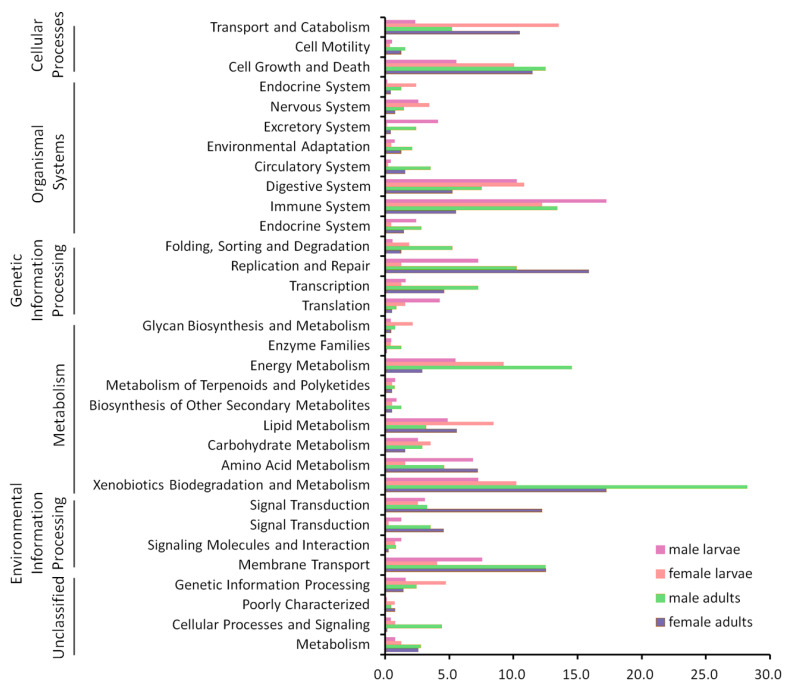
Inferred functions of bacterial communities associated with *P. canidia*. All of the predicted KEGG metabolic pathways are shown at the second hierarchical level and grouped by major functional categories.

We predicted functional potentials of the microbial community associated with the four groups of *P. canidia* (male larvae, female larvae, male adults and female adults) using the PICRUSt (Figure 4), which is a computational approach to predict the functional composition of a metagenome using marker gene data and a database of reference genomes [14]. It is a conventional and effective method to infer the microbiota functional potential across the stages using PICRUSt. These functional categories, including nervous systems and translation, were enriched in the larvae, whereas in the adults, cell motility, circulatory system, transcription, metabolism and associated relative gene copy numbers were increased by approximately 50%. The enrichment of several other pathways, including those for energy metabolism, immune system, digestive system, xenobiotics biodegradation, transport, cell growth and death, were observed in microbiome of all four groups. 

### 3.2. Fitness of P. canidia Treated with Antibiotics

**Figure 5:**
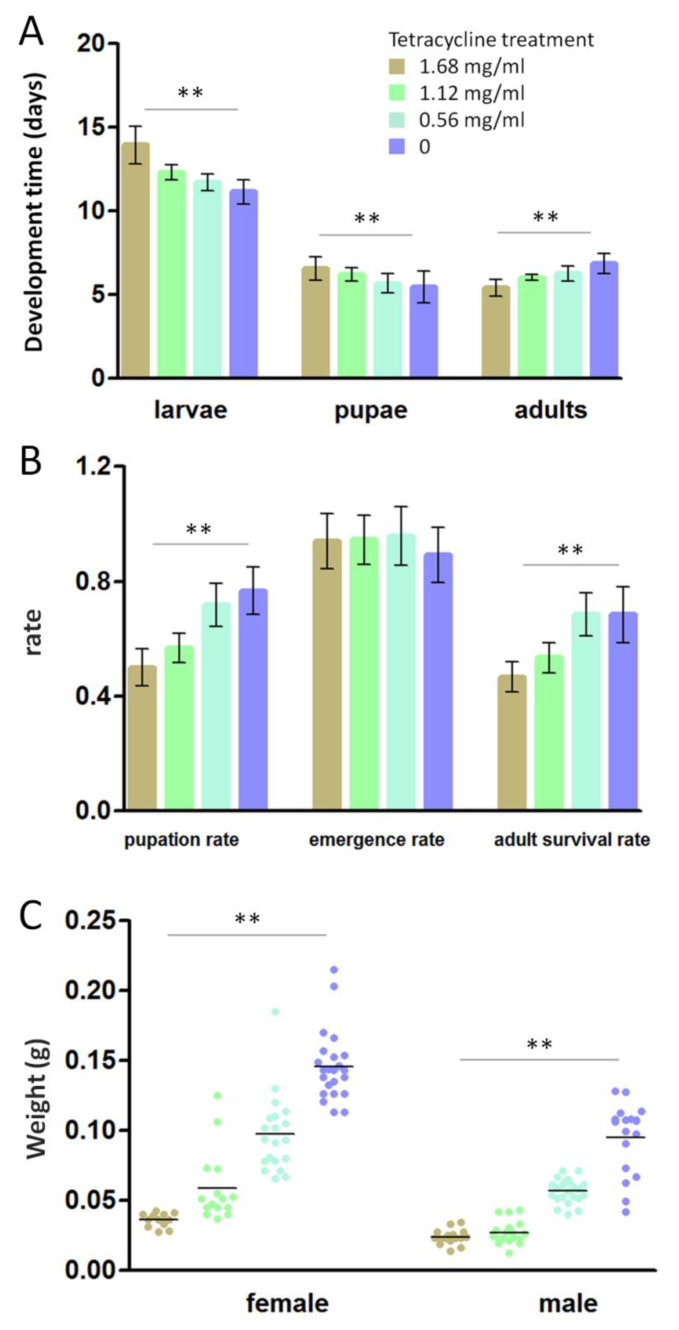
The histograms are for (**A**) development time and (**B**) pupation rate, adult survival rate and emergence rate. The scatter diagram is for (**C**) weight of female and male 5th instars. The bars represent mean ± standard deviation (n = 3), and F tests were used to determine the difference across different concentration of tetracycline treatment (* *p* < 0.05, ** *p* < 0.01).

The data of insect fitness parameters (development time, pupation rate, emergence rate, adult survival rate and weight of 5th instars larvae) are showed in Table 4. For development time, the ANOVA showed a significant difference among three tetracycline treatment groups (0.56 mg/mL, 1.12 mg/mL and 1.68 mg/mL) and the untreated group (Figure 5A). The larvae and pupae with tetracycline treatment had a longer development time than those untreated (F _(3131)_ = 92.92, *p* < 0.01 and F _(3138)_ = 18.09, *p* < 0.01). The adults had a shorter life span when they were treated with antibiotics (F _(3137)_ = 55.28, *p* < 0.01).

The pupation rate was lower when the antibiotics were present and decreased with concentration (Figure 5B), resulting in a significant effect of the antibiotic condition on this variable (F _(3,20)_ = 19.74, *p* < 0.01). A similar significant effect was observed on the adult survival rate (F _(3,30)_ = 13.87, *p* < 0.01). The emergence rate was higher when treated with antibiotics but showed no significant difference from those untreated (F _(3,20)_ = 0.55, *p* = 0.66). 

The weights of female and male adults were significantly higher than those treated with antibiotics (F _(3,66)_ = 70.65, *p* < 0.01 and F _(3,71)_ = 95.95, *p* < 0.01). The weight was lower when the antibiotics were present and increased with concentration (Figure 5C).

## 4. Discussion

The composition of gut microbiomes in lepidopterans is relatively simple [20,21,22]. In this study, the 10 most abundant phylotypes accounted for 74% of the sequences from all *P. canidia* samples, which showed an uneven structure of the bacterial communities. Hammer et al. [7] and Ravenscraft et al. [8] reported that caterpillars lack a resident gut microbiome, and the gut microbial community composition varies across adult butterflies. The high pH values (>10) in gut [23], host-encoded digestive and detoxification peptides [24], simple gut structure of tube-like morphology [25] and fast food transit times may be unfavorable to microbial growth. 

Despite the relatively simple composition and low densities of lepidopteran gut microbiomes [7,20,21,22], this study points to potential fitness advantages with the gut microbiomes in *P. canidia*. The fitness advantages relate to a faster development time, a longer life span of adults, higher pupation and adult survival rates and a heavier weight (Figure 5). After the butterflies were treated with difference concentration of tetracycline (0.56 mg/mL, 1.12 mg/mL and 1.68 mg/mL), these parameters, which are often used as a proxy of insect fitness, showed a significant difference among the treatment group and untreated group.

We characterized gut microbial abundance and composition of the larvae and adults via 16S rRNA gene sequencing and found some abundant phylotypes present across all larvae and adult butterflies samples. The isolate in the genus *Enterobacter* is the phylotype with the highest abundance across all *P. canidia* samples. *Enterobacter* are found to have cellulolytic activity to degrade carbohydrates and may be useful for digestion by the host insect [26]. The cellulose degrading bacteria are suggested to have the ability to utilize xylan, which is a polymer made of β-1, 4 xylosidic bonds [27]. Potrikus and Breznak [28] reported *Enterobacter agglomerans*, isolated from guts of wood-eating termites, has nitrogen-fixing activity and may be important for the nitrogen economy of the termites. 

The other three genera of bacteria examined from all *P. canidia* samples were *Acinetobacter*, *Aquabacterium* and *Rhodococcus*. The denitrifying genus *Acinetobacter* and *Aquabacterium* are commonly present in other insects. They have been shown to confer nutritional benefits including nitrogen removal and intestinal nutrient substance metabolism [29,30,31]. The genus *Rhodococcus* plays an important role in the degradation of toxins, such as monoterpene and isoprene, which is harmful to insect fitness [32]. 

A phylotype matching a *Stenotrophomonas* clone was also abundant. The genus contains species ranging from common soil organisms (*S. nitritireducens*) to opportunistic human pathogens (*S. maltophilia*) [33]. *Stenotrophomonas* sequences have been reported from other lepidopteran species including *Plodia interpunctella* [34] and *Antheraea assamensis* [35], although their possible role in host herbivory is not well understood. Wang et al. [36], reported a strain of *S. maltophilia* from the mesophilic microbial community BYND-8 to be cellulolytic.

The genera *Staphylococcus* and *Pseudomonas*, which are a group of pathogenic bacteria, may cause host insect diseases. When silkworm larvae were fed on mulberry leaves smeared with a bacterial solution of *Staphylococcus*, the gene expression of gloverin and lysozyme was upregulated. When silkworm larvae were fed on mulberry leaves smeared with a bacterial solution of *Pseudomonas*, the gene expression of gloverin 2 and gloverin 3, as well as the genes of the immune related pathway 9 were upregulated. This indicates that both genera are closely related to the immune signaling pathway of the insect [37]. *Rickettsia* can affect the fitness of their hosts, especially under stress. Himler et al. [38] found that compared with uninfected whiteflies, *Rickettsia*-infected whiteflies produced more offspring, had higher survival to adulthood, developed faster and produced a higher proportion of daughters.

## 5. Conclusions

This study used 16S rRNA gene sequencing to clarify the gut microbiome across the life stages and sexes of *P. canidia* and compared the fitness of butterflies treated and untreated with antibiotics. These results suggested that the gut microbiota of *P. canidia* was relatively simple but played a potential role in fitness advantage for its host. The gut bacteria are useful for digestion, nutrient substance metabolism, toxin degradation and immune signaling pathway activity.

## Figures and Tables

**Table 1:** Dominant bacterial phylotypes in *P. canidia*.

Taxonomic Classification	Total	Male Larvae	Female Larvae	Male Adults	Female Adults	Genus/Species Name
Proteobacteria (Enterobacteriaceae)	23.4	1.2	22.5	25.8	45	*Enterobacter*
Proteobacteria (Enterobacteriaceae)	16.8	54.4	0.6	6.1	0.6	*Providencia vermicola*
Proteobacteria (Moraxellaceae)	9.9	8.9	0.5	20	8.9	*Acinetobacter*
Proteobacteria (Moraxellaceae)	7.4	0.01	0.4	19.9	8.8	*Acinetobacter guillouiae*
Proteobacteria (Burkholderiaceae)	3.9	0.01	0.02	16.1	0.06	*Ralstonia mannitolilytica*
Proteobacteria (Enterobacteriaceae)	3.7	9.8	3.9	0.02	0.01	*Kluyvera*
Proteobacteria (Pseudomonadaceae)	3.2	0.5	2.4	9.6	0.9	*Pseudomonas*
Proteobacteria (Xanthomonadaceae)	2.6	0.01	0.6	7.3	1.3	*Stenotrophomonas*
Proteobacteria (Brucellaceae)	1.8	0.02	7.6	0.02	0.3	*Ochrobactrum pseudogrignonense*
Firmicutes (Paenibacillaceae)	1.3	0.01	0.1	0.01	6.8	*Brevibacterium*
Proteobacteria (Rickettsiaceae)	1.1	0.05	1.2	2.7	0.3	*Rickettsia*
Proteobacteria (Comamonadaceae)	0.6	0.01	0.02	2.4	0.08	Uncultured beta proteobacterium
Proteobacteria (Pseudomonadaceae)	0.6	0.01	0.03	2.3	0.3	*Pseudomonas aeruginosa*
Firmicutes (Staphylococcaceae)	0.6	0.01	0.04	0.1	3.6	*Staphylococcus*
Actinobacteria (Nocardiaceae)	0.5	0.2	0.5	0.7	0.6	*Rhodococcus*
Proteobacteria (Pseudomonadaceae)	0.5	0.4	1.6	0.02	0.02	*Pseudomonas protegens*
Firmicutes (Bacillaceae)	0.2	0.01	0.01	0.7	0.01	*Anoxybacillus*
Proteobacteria (Comamonadaceae)	0.2	0.02	0.05	0.2	0.7	*Aquabacterium*
Bacteroidetes (Sphingobacteriaceae)	0.2	0.01	0.02	0.02	1.0	*Sphingobacterium*
Bacteroidetes (Bacteroidaceae)	0.1	0.01	0.02	0.01	0.6	*Bacteroides*

**Table 2:** Samples and their sequencing data processing.

Sample Name	Clean Reads	Tags	OTUs
Male larva 1	30,832	28,467	78
Male larva 2	31,836	29,642	89
Male larva 3	35,873	33,895	40
Female larva 1	30,150	24,244	128
Female larva 2	25,145	23,521	45
Female larva 3	25,612	23,003	48
Male adult 1	38,990	36,377	86
Male adult 2	26,316	24,431	44
Male adult 3	27,648	24,893	82
Female adult 1	47,496	20,165	69
Female adult 2	46,537	15,318	62
Female adult 3	26,431	24,109	80

**Table 3:** Bacterial alpha diversity of *P. canidia* in different groups based on the 16S rDNA amplicon.

	Sobs (Observed Species)	Chao	Ace	Shannon	Simpson
Male larvae	69 ± 5.71	84.85 ± 17.65	103.53 ± 15.19	0.93 ± 0.51	0.56 ± 0.27
Female larvae	73.67 ± 7.08	97.5 ± 7.08	103.94 ± 6.53	1.10 ± 0.55	0.53 ± 0.08
Male adults	70.67 ± 23.18	88.73 ± 30.36	95.71 ± 9.06	1.53 ± 0.35	0.34 ± 0.12
Female adults	70.33 ± 9.07	74.22 ± 11.19	77.21 ± 12.68	2.16 ± 0.94	0.28 ± 0.08

**Table 4:** The data of insect fitness parameters.

Tetracycline Treatment	Group	Development Time (Days)			Weight of 5th Instars Larvae (g)	Pupation Rate	Emergence Rate	Adult Survival Rate
		Larvae	Pupae	Adults				
1.68 mg/mL	1	16	7	6	0.0401	0.50	1.00	0.50
		13	7	5	0.0241			
		15	5	6	0.0234			
		14	7	5	0.0331			
		13	7	6	0.0215			

## Data Availability

The raw sequence data obtained in this study were deposited in the Bioproject of The National Center for Biotechnology Information (NCBI) under accession number PRJNA595480 (https://www.ncbi.nlm.nih.gov/bioproject/PRJNA595480). All authors approved the disclosure of data because this study did not involve human subjects.

## Supplementary Materials

The following are available online at https://www.mdpi.com/2075-1729/10/11/254/s1, Figure S1: Petal diagram of OTU distribution across P. canidia life stages and sexes. Numbers within compartments indicate OTU counts according to mathematical sets.; Figure S2: Petal diagram of OTU distribution across all samples. Numbers within compartments indicate OTU counts according to mathematical sets.; Figure S3: Rarefaction curve based on OTUs. Mothur (v1.31.2) was used to calculate indices for rarefaction curve based on observed species values.
