# Supplementary material for: Diversity, Composition and Functional Inference of Gut Microbiota in Indian Cabbage white Pieris canidia (Lepidoptera: Pieridae)
Source: Life (Basel). 2020 Oct 25;10(11):254. doi: 10.3390/life10110254 (PMC7692319; doi:10.3390/life10110254)
Supplement: Supplementary file 1 [file life-10-00254-s001.pdf]

life

Supplementary Materials of Diversity, Composition and Functional Inference of Gut Microbiota in Indian Cabbage white *Pieris canidia* (Lepidoptera: Pieridae)

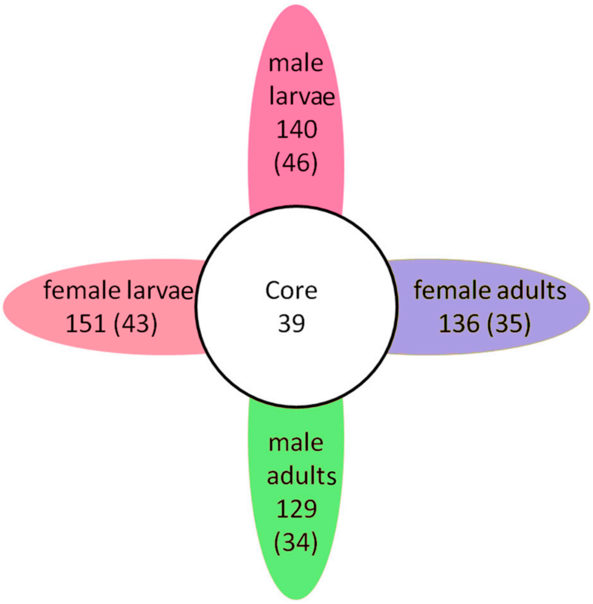

Figure S1. Petal diagram of OTU distribution across *P. canidia* life stages and sexes. Numbers within compartments indicate OTU counts according to mathematical sets.

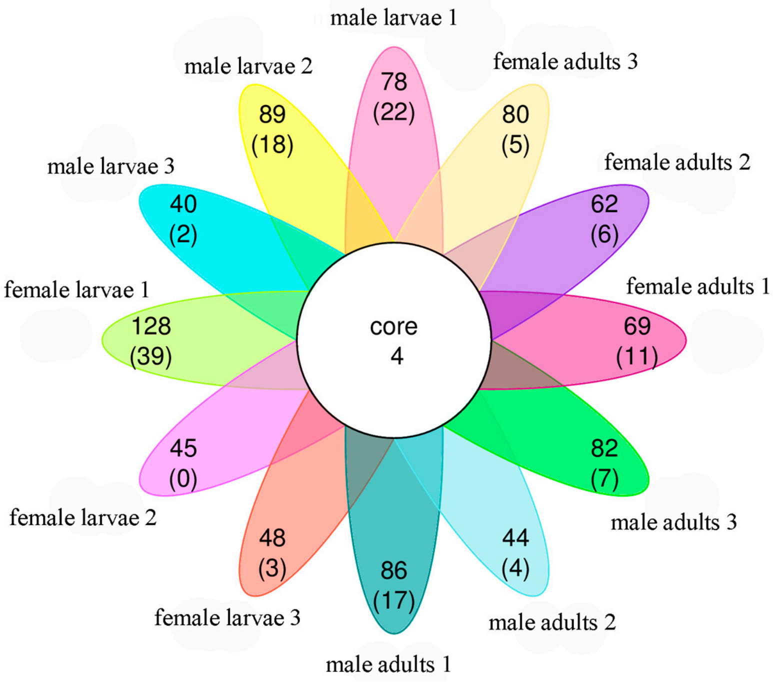

Figure S2. Petal diagram of OTU distribution across all samples. Numbers within compartments indicate OTU counts according to mathematical sets.

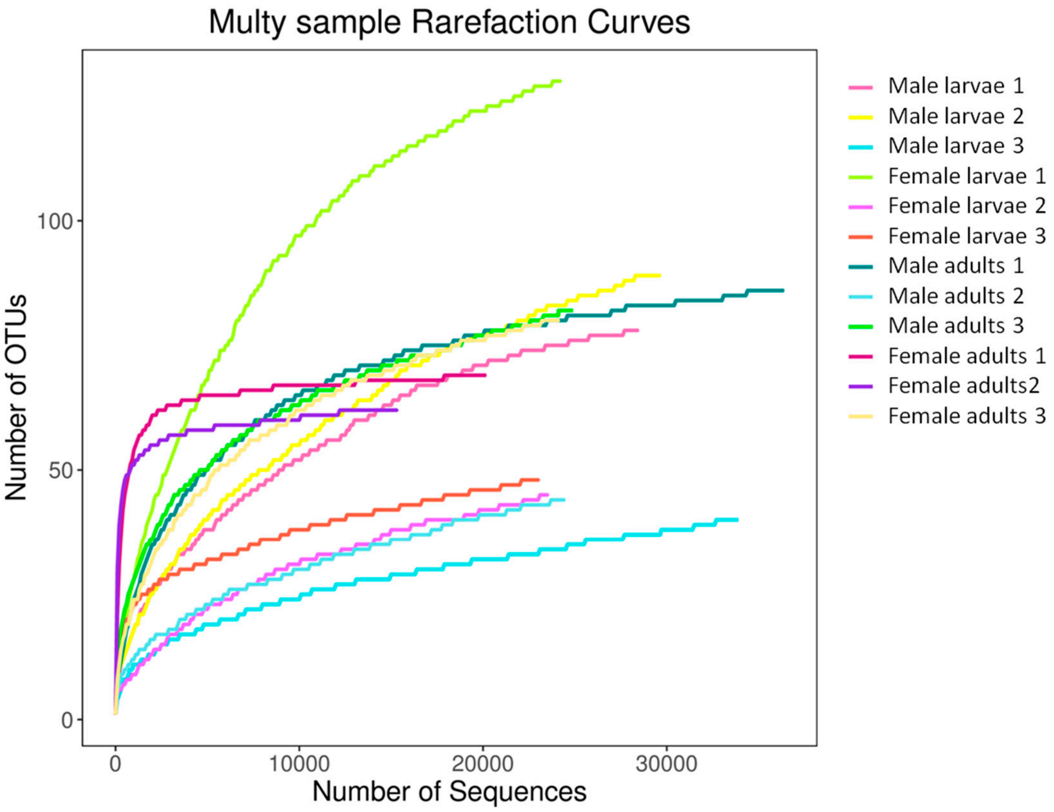

Figure S3. Rarefaction curve based on OTUs. Mothur (v1.31.2) was used to calculate indices for rarefaction curve based on observed species values.

Publisher's Note: MDPI stays neutral with regard to jurisdictional claims in published maps and institutional affiliations.

© 2020 by the authors. Licensee MDPI, Basel, Switzerland. This article is an open access article distributed under the terms and conditions of the Creative Commons Attribution (CC BY) license (http://creativecommons.org/licenses/by/4.0/).
